# Caffeic acid ameliorates colitis in association with increased *Akkermansia* population in the gut microbiota of mice

**DOI:** 10.18632/oncotarget.9306

**Published:** 2016-05-11

**Authors:** Zhan Zhang, Xinyue Wu, Shuyuan Cao, Li Wang, Di Wang, Hui Yang, Yiming Feng, Shoulin Wang, Lei Li

**Affiliations:** ^1^ Department of Hygiene Analysis and Detection, School of Public Health, Nanjing Medical University, Nanjing, Jiangsu, P. R. China; ^2^ Key Laboratory of Modern Toxicology of Ministry of Education, School of Public Health, Nanjing Medical University, Nanjing, P. R. China

**Keywords:** ulcerative colitis, caffeic acid, dextran sulfate sodium, NF-κB pathway, fecal microbiota, Immunology and Microbiology Section, Immune response, Immunity

## Abstract

Emerging evidence shows that dietary agents and phytochemicals contribute to the prevention and treatment of ulcerative colitis (UC). We first reported the effects of dietary caffeic acid (CaA) on murine experimental colitis and on fecal microbiota. Colitis was induced in C57BL/6 mice by administration of 2.5% dextran sulfate sodium (DSS). Mice were fed a control diet or diet with CaA (1 mM). Our results showed that dietary CaA exerted anti-inflammatory effects in DSS colitis mice. Moreover, CaA could significantly suppress the secretion of IL-6, TNFα, and IFNγ and the colonic infiltration of CD3^+^ T cells, CD177^+^ neutrophils and F4/80^+^ macrophages via inhibition of the activation of NF-κB signaling pathway. Analysis of fecal microbiota showed that CaA could restore the reduction of richness and inhibit the increase of the ratio of *Firmicute* to *Bacteroidetes* in DSS colitis mice. And CaA could dramatically increase the proportion of the mucin-degrading bacterium *Akkermansia* in DSS colitis mice. Thus, CaA could ameliorate colonic pathology and inflammation in DSS colitis mice, and it might be associated with a proportional increase in *Akkermansia*.

## INTRODUCTION

Ulcerative colitis (UC) is one of the most common chronic inflammatory bowel diseases (IBD) characterized by persistent progressive or relapsing inflammation, bloody diarrhea, and abdominal distress. Although the etiology of IBD remains unclear, it is thought to result from dysfunction of the mucosal immune responses to intestinal bacterial antigens in genetically predisposed individuals [1, 2]. Currently, 5-Amninosalicylates, antimicrobials, steroids, and immune modulators have been used to suppress mucosal immune responses [3]. Unfortunately, long-term usage of these agents has been found to lead to severe toxicities, highlighting the need for novel therapeutic targets [4].

The chemically induced dextran sulfate sodium (DSS) colitis model has been shown to mimic human UC pathology, and recent preclinical studies have supported its use as a system to evaluate the role of anti-inflammatory agents [5, 6]. Epidemiologic studies of IBD indicate that tea/coffee consumption is a protective factor for UC in Asia [7]. Coffee is a major source of chlorogenic acid in the diet with a daily intake of about 1g in coffee drinkers. Caffeic acid (CaA) is a hydrolyzed metabolite of chlorogenic acid by mucosal and/or microbial esterase in the intestinal tract [8]. In addition, CaA is found in argan oil, oats, wheat, rice and olive oil [9]. The anti-inflammatory effect of CaA on DSS-induced experimental colitis was reported in C3H/HeOuJ mice [10]. However, the mechanism of the amelioration of acute colitis by CaA is still unclear.

The intestinal microbial dysbiosis contributes to the development of UC and in determining subsequent disease behavior and outcomes [11]. *Akkermansia* is a Gram-negative, strict anaerobe belonging to *Verrucomicrobia* and it is a mucin-degrading bacterium that lives in the mucus layer of the intestine and represents 1- 3% of the total gut microbiota [12]. There has also been a growing interest in *Akkermansia* due to its association with health in animals and humans. Notably, reduced levels of *Akkermansia* have been observed in patients with IBD (mainly UC) and metabolic disorders, which suggests it may have potential anti-inflammatory properties [13]. Dietary polyphenols promote growth of the gut bacterium *Akkermansia* and attenuate high fat diet-induced metabolic syndrome [14]. As a kind of phenolic phytochemicals, the effect of CaA on *Akkermansia* is still unknown. The aim of the present study was to define the influence of CaA on DSS-induced colitis and gut microbiota.

## RESULTS

### Dietary CaA improved the disease activity index (DAI) of mice treated with DSS

**Figure 1:**
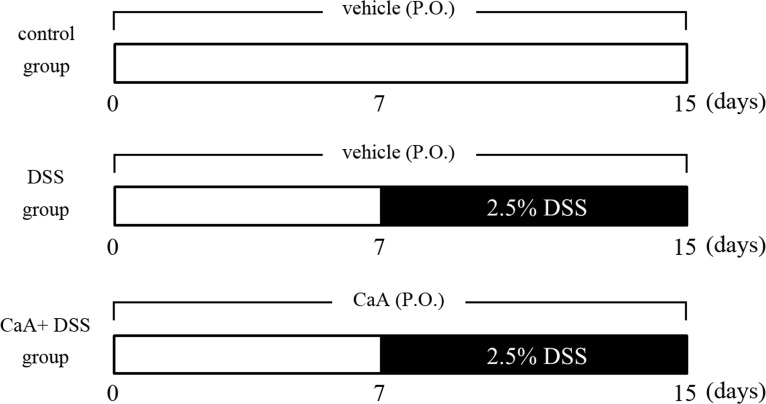
Schematic diagram of the experimental study design The mice were divided into 3 groups: control, DSS and CaA+ DSS. The control group was given autoclaved water for 15 days; the other 2 groups were given autoclaved water for the first 7 days, and then given water containing 2.5% DSS for the last 8 days. The CaA+ DSS group was orally administered with 1 mM CaA for 15 days.

**Figure 2:**
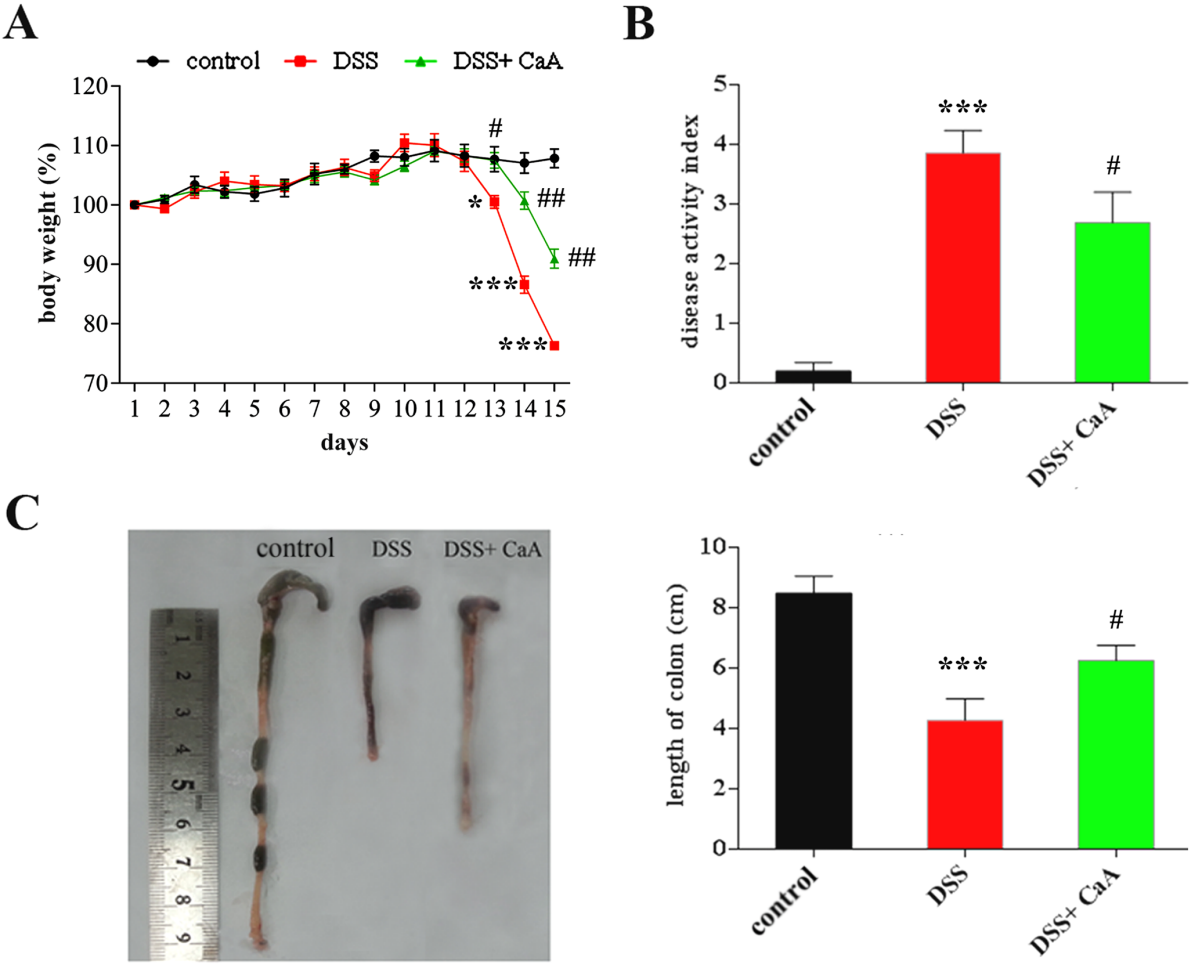
Dietary CaA improved DSS-induced colitis in mice **A.** Data for weight changes are expressed as the mean percentage change from the starting body weight. **B.** Disease activity index was evaluated as average of score of clinical parameters as body weight changes, rectal bleeding and stool consistency or diarrhea. **C.** Colon length of each group. The data are expressed as the mean ± SD from 10 mice in each group. *P < 0.05, ***P < 0.001, compared with control group; ^#^*P* < 0.05, ^##^*P* < 0.01, ^###^*P* < 0.001, compared with DSS group.

A schematic diagram of the experimental study design was shown in Figure 1. Loss of body weight, apparent diarrhea, and rectal bleeding are symptoms present in all DSS-treated mice. Significant loss of body weight was observed from the fifth day of DSS treatment, CaA treatment could recover this loss in body weight (Figure 2A). Compared with the control, the increased DAI (Figure 2B) and shortening of the colon (Figure 2C) were observed in DSS-treated mice, CaA supplementation could significantly ameliorate these effects.

### Histopathological analysis of DSS-induced acute colitis after CaA supplementation

**Figure 3:**
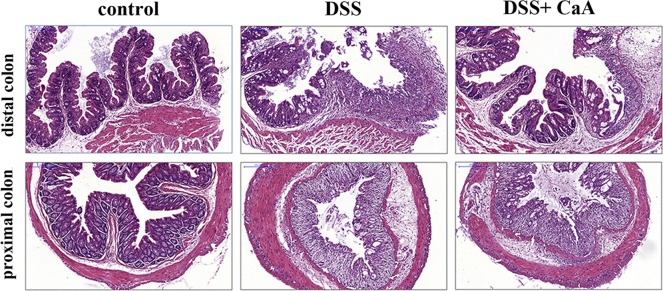
Effects of CaA on the histopathological characterization in DSS-induced mouse colitis Representative images of the proximal and distal colonic tissues from control, DSS and DSS+ CaA group. Formalin fixed, paraffin-embedded 5 μm cross-sections were stained with H&E. Scale bar: 50 μm.

Compared with the control, mice treated with DSS exhibited serious injuries that affected both the proximal and distal colon, loss of histological structure, strong epithelial disintegration, disruption of the epithelial barrier, a pronounced decrease in the number of crypts, and marked infiltration of inflammatory cells into the mucosa and submucosa (Figure 3). In contrast, colonic slides from the DSS+ CaA group revealed reduced signs of inflammation into the colonic tissue and a minor extent affected mucosa with moderate loss of epithelial cells, especially in distal colonic segments (Figure 3).

### Effects of CaA on serum cytokines and colonic infiltration of inflammatory cells in DSS-treated mice

**Figure 4:**
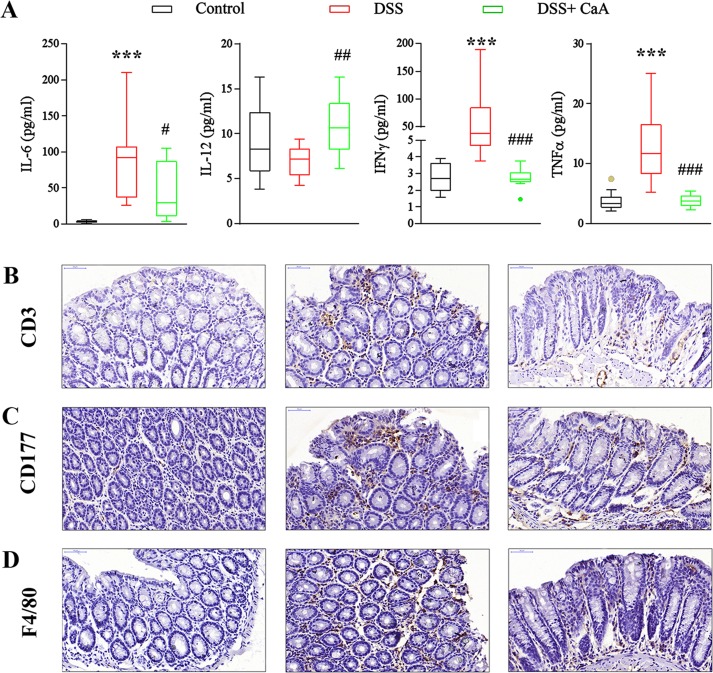
Effects of CaA on serum cytokines and colonic infiltration of inflammatory cells in DSS-colitis mice **A.** The serum concentration of IL-6, IL-12, IFNγ and TNF α were detected, and the statistical difference was analyzed by Mann-Whitney U test. ***P < 0.001, compared with control group; ^#^*P* < 0.05, ^##^*P* < 0.01, ^###^*P* < 0.001, compared with DSS group. Representative images of **B.** CD3, **C.** CD177, and **D.** F4/80 immunostaining in the distal colon of mice 1 week after cessation of DSS treatment. Formalin fixed, paraffin-embedded 5 μm cross-sections were stained with respective primary antibody. Scale bar: 50 μm.

Compared with the control, the serum levels of IL-1β, IL-6, IL-10, IFNγ, and TNFα were increased in DSS colitis mice (Figure 4A and S1). CaA supplementation significantly reduced the serum level of IL-6, IFNγ and TNFα (Figure 4A). In addition, CaA significantly increased the level of IL-12 in the mice treated with DSS. Compared to the control mice, DSS triggered an increased infiltration of CD3^+^ T cells (Figure 4B), CD177^+^ neutrophils (Figure 4C), and F4/80^+^ macrophages (Figure 4D) into the colonic lesion area.

### Effects of CaA on NF-κB signaling in DSS-treated mice

**Figure 5:**
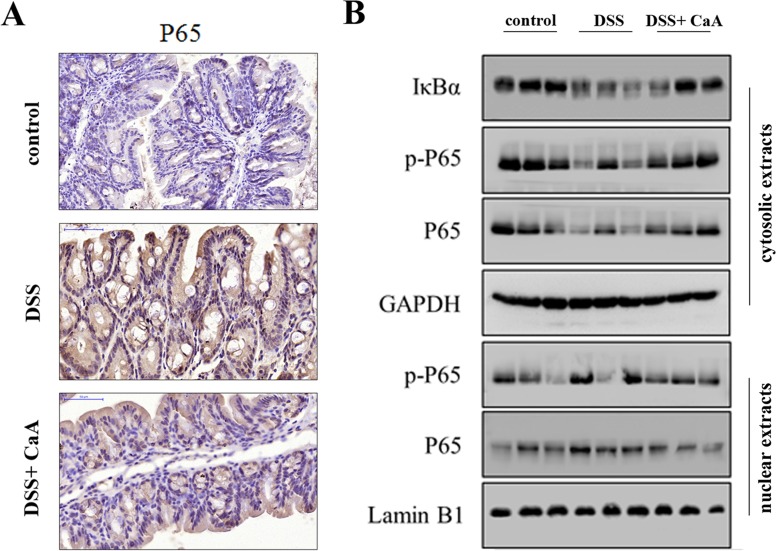
Effects of CaA on NF-κB signaling in DSS-treated mice **A.** Immunohistochemical analysis for P65 in distal colon of mice. Formalin fixed, paraffin-embedded 5 μm cross-sections were stained with P65 primary antibody. Scale bar: 50 μm. **B.** Immunoblotting assay of the expression of NF-κB signaling in nuclear and cytoplasmic fractions of mice colon in each group. Tissue lysates protein (100 μg) was prepared to determine protein expression using an immunoblotting assay with antibodies specific for IκBα, p-P65 and P65 under the same experimental conditions. GAPDH and lamin B1 were used as cytoplasmic and nuclear markers.

DSS treatment caused a significant increase in P65, and P65 abundance in the CaA group was significantly decreased compared to the DSS group (Figure 5A). Furthermore, the expression of cytoplasmic IκBα, phosphorylated P65 (p-P65) and total P65 significantly decreased in response to DSS. Meanwhile, the expression of nuclear p-P65 and total P65 significantly increased. These results suggest that NF-κB signaling was activated by DSS, and CaA treatment could inhibit these effects (Figure 5B).

### Effects of CaA on fecal microbiota in DSS-treated mice

The ACE and Chao indices demonstrated that the richness of fecal microbiota in DSS colitis mice were significantly lower than that in controls, CaA could reverse these effects (Table 1). These effects were confirmed by the rarefaction curves (Figure S2A) and rank abundance curves (Figure S2B). Compared with the control, the Shannon and Simpson indices demonstrated that the fecal microbiota diversity was decreased in DSS colitis mice, CaA exacerbated this reduction (Table 1).

**Table 1:** Comparison of phylotypes coverage and diversity estimation of the 16S rRNA gene libraries for individuals at 97% similarity from the sequencing analysis

Sample	No. of reads^a^	No. of OTUs^b^	Coverage (%)^c^	Richness estimator^d^				Diversity index^d^			
				ACE	95% CI	Chao	95% CI	Shannon	95% CI	Simpson	95% CI
control	147205	299	99.99	304	(301, 315)	306	(301, 324)	4.01	(4.00, 4.02)	0.0345	(0.0342, 0.0348)
DSS	147205	283	99.99	302	(292, 322)	304	(291, 336)	3.35	(3.34, 3.36)	0.0785	(0.0777, 0.0793)
DSS+ CaA	147205	316	99.99	325	(320, 338)	323	(318, 338)	3.25	(3.24, 3.26)	0.1054	(0.1044, 0.1065)

a The samples were rarefied to 147205 reads per group for downstream analysis.

b The operational taxonomic units (OTUs) were defined at the 97% similarity level.

c The coverage percentage was calculated using Good's method.

d The richness estimators (ACE and Chao) and diversity indices (Shannon and Simpson) were calculated using the mothur program.

**Figure 6:**
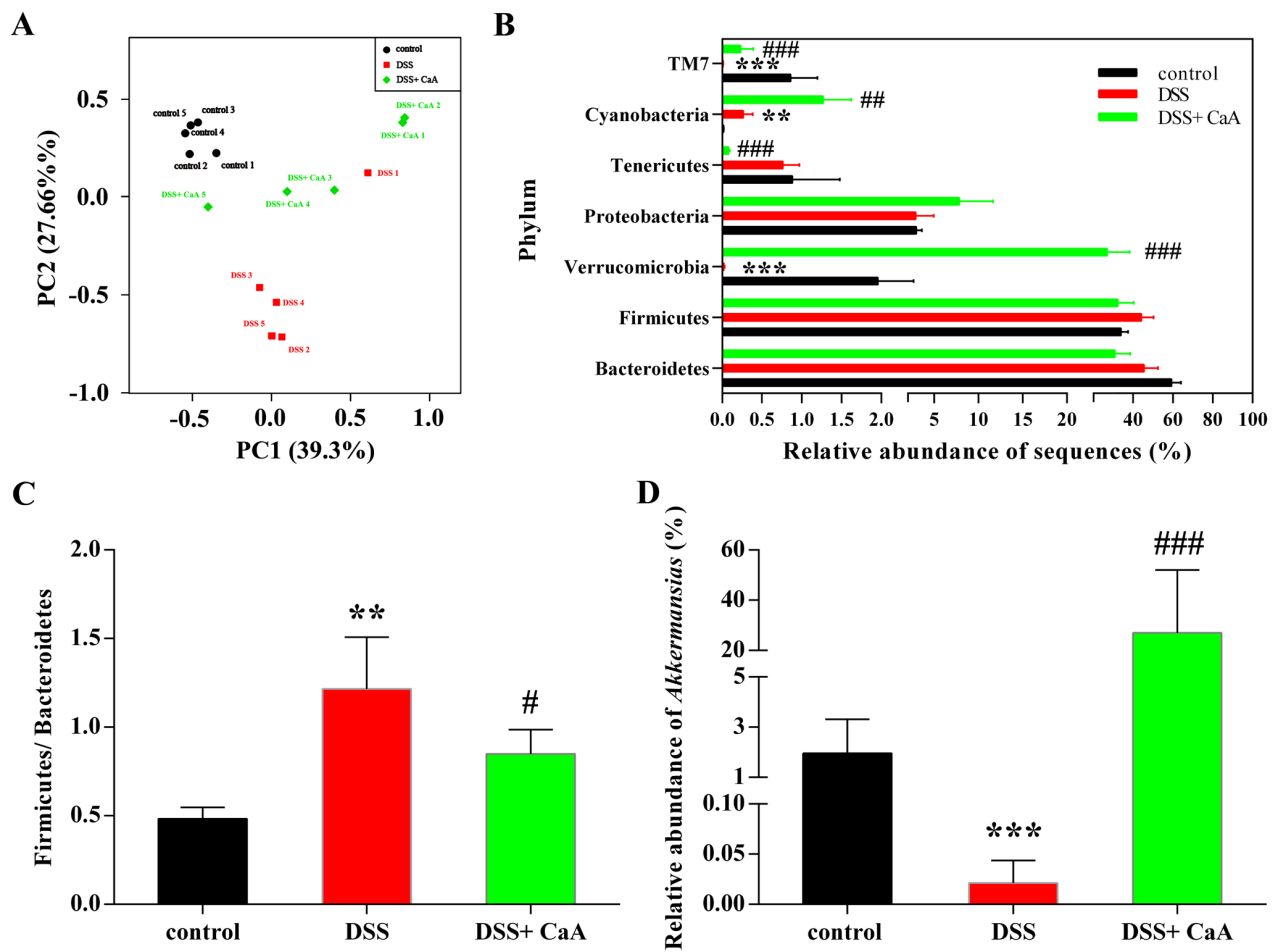
Effects of CaA on the structure of fecal microbiota in DSS colitis mice **A.** Principal coordinate analysis plot of the fecal microbiota based on the Bray-Curtis. **B.** Taxonomic differences of fecal microbiota between DSS and DSS+ CaA groups at the bacterial phylum. **C.** Ratio of the percentage of 16S rRNA gene sequences assigned to *Firmicutes versus Bacteroidetes*. **D.** Comparison of the relative abundance of *Akkermansia* in fecal samples. **P < 0.01, ***P < 0.001, compared with control group; ^##^*P* < 0.01, ^###^*P* < 0.001, compared with DSS group.

Despite significant inter-individual variation, the fecal microbiota from the three groups could be separated clearly by PCoA according to the community composition (Figure 6A). These effects were confirmed by cluster analysis (Figure S2C) and NMDS (Figure S2D). Taxonomic bins at the phylum level showed that the proportion of *Firmicute* or *Bacteroidetes* had no significant changes in DSS colitis mice (Figure 6B). However, the ratio of *Firmicute* to *Bacteroidetes* increased in DSS colitis mice, and CaA could inhibit this effect (Figure 6C). In addition, CaA supplementation significantly reversed underrepresentation of *Verrucomicrobia* in DSS colitis mice (Figure 6B). Further analysis showed that CaA treatment significantly increased the relative abundance of *Akkermansia* in DSS colitis mice (Figure 6D).

## DISCUSSION

Preclinical studies have shown that certain dietary phytochemicals possess beneficial effects in preventing/ameliorating UC [4]. However, the mechanism of the amelioration of acute colitis by CaA is still unclear. Administration of CaA effectively attenuated body weight loss, colon length shorting and macroscopic and histological changes in DSS colitis mice. The DSS colitis model has been characterized by increased serum levels of IL-6, IL-17, and TNFα and elevated levels of IL-4, IL-6, IL-10 and IFNγ have been reported in chronic colitis [15, 16]. In the present study, CaA could significantly reverse the increase of IL-6, TNFα and IFNγ in DSS colitis mice. Increased colonic infiltration of T cells, macrophages, and neutrophils were involved in DSS-induced colitis [17, 18]. And selective blockage of inflammatory mediator decreased neutrophils/ macrophages migration, improving colitis progression [19, 20]. Treatment of CaA may inhibit the colonic infiltration of T cells, macrophages and neutrophil to improve acute colitis.

NF-κB, redox-sensitive transcription factor, is a key regulator of inflammation, innate immunity, and tissue integrity [21]. The nuclear translocation of NF-κB is strongly activated by experimental colitis models, as well as in patients with IBD [22]. In the present study, the NF-κB signaling pathway was significantly activated in DSS colitis mice. The perpetuated activation of NF-κB in patients with active IBD suggests that regulation of NF-κB activity is a very attractive target for therapeutic intervention. Many chemicals, such as gadolinium chloride and porcine β-defensin 2 could ameliorate UC through inhibition of the NF-κB signaling pathway [17, 23]. Consistent with a previous study [24], CaA could suppress the pro-inflammatory response by blocking the activation of NF-κB.

Intestinal microbiota also plays a central role in chronic inflammatory disease, such as UC [25]. Similar to a previous study [26], DSS treatment perturbed the community composition of fecal microbiota. The ratio of *Firmicutes* and *Bacteroidetes* significantly increased in DSS colitis mice, while treatment of CaA attenuated this increase. First, the present study reported that CaA could restore the reduction of richness of fecal microbiota in DSS colitis mice. Dietary polyphenols in instances such as CaA may contribute to the maintenance of intestinal health by preserving the gut microbial balance through the stimulation of the growth of beneficial bacteria and the inhibition of pathogenic bacteria [27]. However, the mechanisms by which CaA exert probiotic effects and reshaping of the gut microbiota which benefits the host are still unclear.

*Akkermansia* is a gram-negative anaerobe belonging to *Verrucomicrobia*, it can degrade intestinal mucins, the highly glycosylated proteins of epithelial mucus layer, as its sole source of carbon and nitrogen [28]. Emerging evidences show that reduced numbers of *Akkermansia* have been detected in IBD (mainly UC) patients both in clinically active disease and during remission as compared to healthy individuals [29, 30]. In the present study, CaA could reverse the reduction of *Akkermansia* in DSS colitis mice. Extracellular vesicles derived from *Akkermansia* could protect the progression of DSS-induced colitis [31]. It has been reported that *Akkermansia* administration can re-establish the mucus layer integrity in diet-induced obese mice [32], it is possible that a direct trophic effect of CaA on *Akkermansia* precedes the positive effects found on the mucus layer integrity.

Emerging evidence shows that there is a mechanistic link between polyphenols and *Akkermansia* [33]. Administration of green tea or grape polyphenols could increase the proportion of *Akkermansia* in high fat fed mice [14, 34]. *Akkermansia* can display a rapid growth rate in order to monopolize the resources when competition is low in instances such as gastric bypass surgery, and antibiotic therapy [35] CaA has been shown to possess antibacterial activity [36], which could be associated with a reduction in the abundance of species capable of holding *Akkermansia*, thus favouring a rise in its proportion. In addition, our previous study had shown that CaA exert antioxidative activity [37], and its strong oxygen radical scavenging capacity may provide a survival advantage for *Akkermansia*. However, we have not directly established the causal relationship between the increase in the relative proportion of *Akkermansia* populations and the amelioration of colitis.

In summary, the present study demonstrated that dietary CaA could ameliorate DSS-induced acute colitis, resulting in an overall attenuation of macroscopic and histological changes. Inhibition of NF-κB signaling pathways may contribute to the reduction of the infiltration of immune cells and inflammatory cytokine secretions. Our study further suggests that CaA could raise the relative proportion of *Akkermansia*, which may be in association with this protective effect.

## MATERIALS AND METHODS

### Ethics statement

This investigation has been conducted in accordance with the ethical standards and according to the Declaration of Helsinki and according to national and international guidelines and has been approved by the Animal Care and Use Committee of Nanjing Medical University.

### Animal treatment

Female C57BL/6 mice (18~20 g) were obtained from Shanghai SLAC Laboratory Animal Co., Ltd (Shanghai, China). The mice were allowed to acclimate for 1 week before the study began. The mice were divided into 3 groups, 10 mice in each group. The control group was given autoclaved water for 15 days; the DSS group was given autoclaved water for first 7 days, and then given water containing 2.5% DSS (molecular weight of 36~50 kDa, MP Biomedicals Solon, OH, USA) for the last 8 days; the DSS+ CaA group was given water containing 1 mM CaA (purity≥ 98.0%, Sigma, St. Louis, MO) for 15 days, and the water also contained 2.5% DSS since the 8th day (Figure 1). Body weight was measured daily. DSS colitis was scored as the disease activity index (DAI) as described previously [38]. In brief, the DAI was the combined scores of weight loss (0, none; 1, 0-5%; 2, 5-10%; 3, 10-20%; and 4, >20%), stool consistency change (0, none; 2, loose stool; and 4, diarrhea), and bleeding (0, none; 1, trace; 2, mild hemoccult; 3, obvious hemoccult; and 4, gross bleeding), and then divided by three. The animals were scored for the DAI at the same time of each day, blind to the treatment. The minimal score was 0 and the maximal score was 4.

The blood samples were drawn orbitally and allowed to clot at room temperature for 2 h before centrifugation (3,000 g, 4°C, 10 min), and the serum was collected and stored at −80°C until use. At necropsy, colons were excised, and the length was measured from the ileocecal junction to the anus. Then, they were rinsed in PBS, and divided into three segments of equal length (proximal, middle, and distal). Sections from proximal (1~2 cm from the cecum) and distal (1~2 cm from the anal verge) segment were fixed in 10% neutral buffered formalin, paraffin embedded, and stained with hematoxylin and eosin for examination by Pannoramic digital slide scanners (Pannoramic SCAN, 3DHISTECH Kft, Budapest, Hungary).

### Immunohistochemistry and immunoblotting assay

The immunohistochemistry and immunoblotting assays were as described in our previous study [39]. The expression of CD 3, CD77, F4/80 and P65 were determined by immunohistochemistry assay. The expression of IκBα, P65 and p-P65 were determined by immunoblotting assay. GAPDH and lamin B1 were used as cytoplasmic and nuclear markers. Antibodies specific for CD3, CD177, F4/80 and GAPDH were obtained from Santa Cruz Biotechnology (Santa Cruz, CA). Antibodies specific for IκBα, P65, p-P65, lamin B1 and an enhanced chemiluminescence (ECL) immunoblotting assay kit were purchased from Cell Signaling Technology (Danvers, MA).

### Multiplex serum cytokine profiling

For quantitative analysis of cytokines (IL-1β, IL-2, IL-4, IL-5, IL-6, IL-10, IL-12, IL-17, TNF-α, and IFN-γ), serum samples were thawed and all candidate cytokines were measured using MILLIPLEXTM micro-beads arrays (Millipore, Billerica, MA) run on a Luminex MAPIG instrument following the manufacturer's recommended protocols [40].

### Microbial DNA extraction

10 mice in each group were randomly divided into 5 cages on day 14 and the stools from one cage were collected as one sample on day 15 before sacrifice. Bacterial genomic DNA from fecal samples (~500 mg) was extracted using QIAamp○RR DNA Stool Mini Kit (Qiagen, Hilden, Germany) according to the manufacturer's instructions.

### Bioinformatics

Bacterial 16S rRNA at the V3 hypervariable region was amplified using a set of primers (338F: 5′-GTGCCAGCMGCCGCGGTAA-3′ and 806R: 5′- GGACTACHVGGGTWTCTAAT-3′). Sequencing was performed by an Illumina MiSeq (PE300). Sequences were then trimmed and classified with the QIIME toolkit. The high-quality reads were clustered into operational taxonomic units (OTUs) using Mothur. The OTUs that reached a 97% nucleotide similarity level were used for alpha diversity (Shannon and Simpson index), richness (ACE and Chao1), Good's coverage, and rarefaction curve analysis using Mothur. Principal coordinate analysis (PCoA), dendrogram and nonmetric multidimensional scaling (NMDS) were performed using OTUs for each sample by Bray-Curtis. Taxonomy-based analyses were performed by classifying each sequence using the Naïve Bayesian Classifier program of the Michigan State University Center for Microbial Ecology Ribosomal Database Project (RDP) database (http://rdp.cme.msu.edu/) with a 70% bootstrap score.

### Statistical analysis

The differences in body weight, colon length and DAI were analyzed using one-way analysis of variance (ANOVA) by SPSS 13.0 software (Chicago, IL, USA). A Mann-Whitney U test was used to assess the differences in cytokines and taxonomy of fecal microbiota. A *P* value of less than 0.05 was considered significant.

## SUPPLEMENTARY MATERIAL FIGURES


