# Supplementary material for: Caffeic acid ameliorates colitis in association with increased Akkermansia population in the gut microbiota of mice
Source: Oncotarget. 2016 May 11;7(22):31790–9. doi: 10.18632/oncotarget.9306 (PMC5077976; doi:10.18632/oncotarget.9306)
Supplement: Supplementary file 1 [file oncotarget-07-31790-s001.pdf]

Caffeic acid ameliorates colitis in association with increased *Akkermansia* population in the gut microbiota of mice

Supplementary Material

RESULTS

Effects of CaA on the fecal microbiota in DSS colitis mice

The rarefaction curves also showed that CaA could reverse the decrease of the species richness of the fecal microbiota in DSS-treated mice, although the shape of the curve revealed that the total richness of the microbial community had not been sampled completely (Fig. S2A). The rank abundance curves indicated that CaA could reverse the decrease of the species richness of the fecal microbiota in DSS-treated mice (Fig. S2B). Despite significant inter-individual variation, the fecal microbiota from the three groups could be divided into three different clusters according to the community composition (Fig. S2C), and could be separated clearly by NMDS (Fig.S2D).

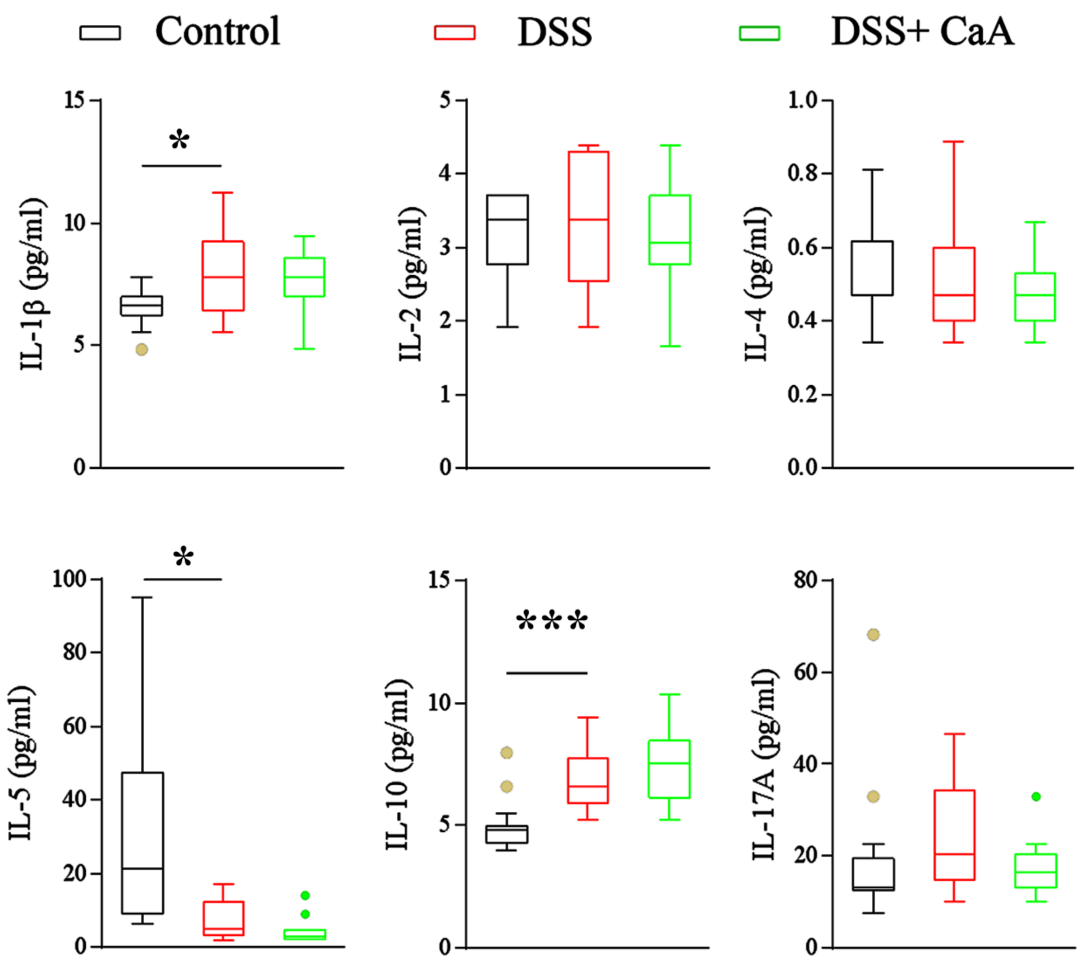

Figure S1. Effects of CaA on serum cytokines in DSS colitis mice. Comparison of the serum concentration of IL-1β, IL-2, IL-4, IL-5, IL-10, and IL-17A were performed by Mann-Whitney U test. *P< 0.05, **P< 0.01, ***P<0.001, compared with control group.

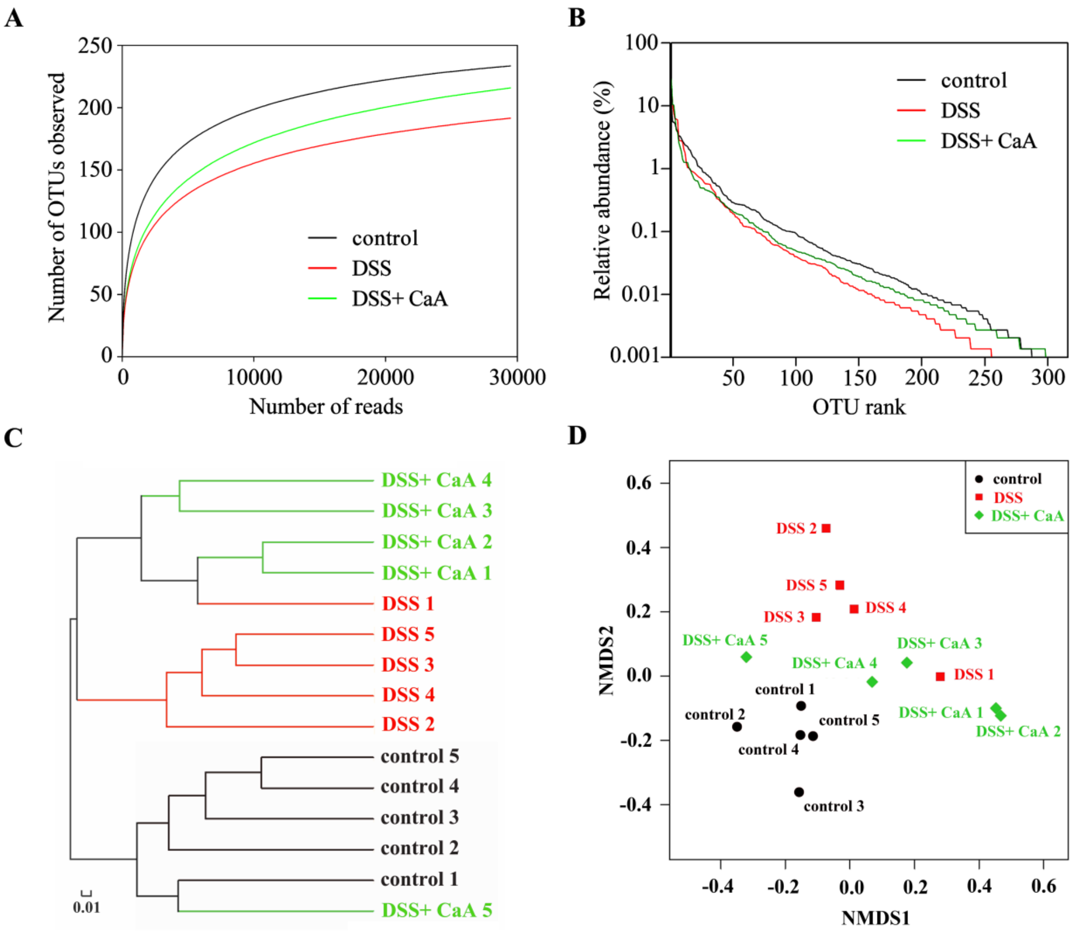

Figure S2. Effects of CaA on the fecal microbiota in DSS colitis mice. (A) Rarefaction curves were used to estimate richness (at a 97% similarity level) of fecal microbiota among the three groups. The X-axis, the number of individual sequences, was recovered in each sample. The Y-axis, as the number of unique OTUs recovered, was determined at an equal sampling effort. **(B)** Rank abundance curve of bacterial OTUs derived from the three groups. **(C)** Dendrogram compares sample pairs using Bray-Curtis similarity indices among controls, DSS and DSS+ CaA groups. The scale bar indicated the distance between clusters. **(D)** Nonmetric multidimensional scaling (NMDS) based on Bray-Curtis among controls, DSS and DSS+ CaA groups.
